# Genetic characterization of Italian patients with Bardet-Biedl syndrome and correlation to ocular, renal and audio-vestibular phenotype: identification of eleven novel pathogenic sequence variants

**DOI:** 10.1186/s12881-017-0372-0

**Published:** 2017-02-01

**Authors:** Gabriella Esposito, Francesco Testa, Miriam Zacchia, Anna Alessia Crispo, Valentina Di Iorio, Giovanna Capolongo, Luca Rinaldi, Marcella D’Antonio, Tiziana Fioretti, Pasquale Iadicicco, Settimio Rossi, Annamaria Franzè, Elio Marciano, Giovanbattista Capasso, Francesca Simonelli, Francesco Salvatore

**Affiliations:** 1 CEINGE-Biotecnologie Avanzate s.c.a r.l., Via Gaetano Salvatore 486, I-80145 Naples, Italy; 2 Department of Molecular Medicine and Medical Biotechnologies, University of Naples Federico II, Via Sergio Pansini 5, I-80131 Naples, Italy; 3 Eye Clinic, Multidisciplinary Department of Medical, Surgical and Dental Sciences, Second University of Naples, Via Sergio Pansini 5, I-80131 Naples, Italy; 4 Department of Nephrology, Second University of Naples, Via Sergio Pansini 5, I-80131 Naples, Italy; 5 Area of Audiology, Department of Neurosciences, Reproductive and Odontostomatological Sciences, University of Naples Federico II, Via Sergio Pansini 5, I-80131 Naples, Italy; 6 IRCCS-Fondazione SDN Naples, Via Emanuele Gianturco 113, I-80143 Naples, Italy

**Keywords:** Bardet-Biedl syndrome, *BBS1*, *BBS2* and *BBS10* gene variants, Ciliopathy, Renal, ocular and audiovestibular phenotype

## Abstract

**Background:**

Bardet-Biedl syndrome (BBS) is a rare genetic disorder that features retinal degeneration, obesity, polydactyly, learning disabilities and renal abnormalities. The diagnosis is often missed at birth, the median age at diagnosis being 9 years. In the attempt to shed light on BBS and improve its diagnosis and treatment, we evaluated the genotype-phenotype relationship in patients with a molecular diagnosis of BBS.

**Methods:**

We analyzed three common BBS genes, *BBS1*, *BBS10* and *BBS2*, in 25 Italian patients fulfilling the clinical criteria of BBS. In 12 patients, we identified gene-specific biallelic variants and thus correlated genotype to the ophthalmic, renal and audio-vestibular phenotypes.

**Results:**

At least one sequence variant was found in 60% of patients. The most common mutated gene was *BBS1* followed by *BBS10*. Of the 17 sequence variants we found, 11 have not previously been associated with BBS. In 12 patients, we identified biallelic pathogenic variants; they had retinitis pigmentosa with early onset of visual impairment. However, retinal dystrophy was less severe in patients with *BBS1* than in those with *BBS10* variants. Overall, we found a high prevalence of renal dysmorphism and dysfunction. Notably, patients with *BBS10* variants had the most severe renal impairment, which resulted in a critical decline in renal function. All the patients who underwent audio-vestibular evaluation had dysfunction of the cochlear outer hair cells, thus confirming the presence of hearing defects.

**Conclusion:**

*BBS1*, *BBS2* and *BBS10* are major causative genes in Italian BBS patients. *BBS10* was associated with the worse outcome in terms of the renal, ocular and audiovestibular phenotypes. Cochlear dysfunction should be included among the hallmarks of BBS.

**Electronic supplementary material:**

The online version of this article (doi:10.1186/s12881-017-0372-0) contains supplementary material, which is available to authorized users.

## Background

Bardet-Biedl syndrome (BBS) is a systemic hereditary disorder characterized by the coexistence of rod-cone dystrophy, polydactyly, obesity, cognitive impairment, and renal dysfunction. Its prevalence varies among geographic areas, ranging from 1:160,000 in North Europe to 1:13,500 in Kuwait and Newfoundland [1]. The phenotype is heterogeneous and the diagnosis is often missed at birth. Polydactyly or syndactyly are generally the first signs recognized at birth, while visual defects, obesity and cognitive impairment develop during the first year of age [2].

Retinal degeneration occurs in over 90% of BBS patients and visual prognosis is poor [3]. Retinal dystrophy in BBS is progressive and varies in severity. Patients experience progressive night blindness, followed by photophobia and loss of central and color vision. At clinical level, they show marked reduction of electroretinogram (ERG) amplitude, which depends on a primary loss of rod photoreceptors followed by cone death [4]. An in vivo micro-structural analysis of retinal layers in patients with BBS revealed macular involvement [5]. The prevalence of renal impairment varies among studies, which however ultimately indicated that few BBS patients have a reduced glomerular filtration rate (GFR), while most have an abnormal renal structure and a normal GFR [2, 6]. Whether an abnormal kidney structure predisposes to progressive renal failure remains to be elucidated. Interestingly, hearing loss is not considered a main feature of BBS, and has been reported in only two studies [7, 8].

BBS is considered an autosomal recessive disease characterized by genetic heterogeneity, which at least partly explains the clinical variability of this condition, although oligogenic inheritance has also been hypothesized [1, 9, 10]. Indeed, biallelic mutations have been reported in 19 genes, namely *BBS1*, *BBS2*, *ARL6*, *BBS4*, *BBS5*, *BBS6 (MKKS)*, *BBS7*, *BBS8 (TTC8)*, *BBS9*, *BBS10*, *BBS11 (TRIM32)*, *BBS12*, *MKS1*, *CEP290*, *C2ORF86*, *SDCCAG8*, *LZTFL1*, *BBIP1*, *IFT27*, which account for 70–80% of BBS cases [11, 12]. The various BBS-associated genes encode proteins involved in the regulation of ciliary structure, biogenesis and function [1]. Therefore, cilia dysfunction is the main hypothesis of BBS pathogenesis [1]. In this context, it is notable that BBS shares several characteristic features with such other ciliopathies as Meckel, Joubert, and Senior-Locken syndromes.

Here, we report the genetic and clinical findings (ocular, renal and audio-vestibular phenotypes) in a cohort of Italian patients who fulfilled the diagnostic criteria of BBS. We focused the molecular analysis on the most common disease-associated genes in Caucasians, namely, *BBS1*, *BBS10* and *BBS2* [13]. Our study aims to expand the spectrum of pathogenic variants associated to BBS in Italy and, by analyzing genotype-phenotype correlations, to improve the diagnosis and treatment of this complex genetic disorder.

## Methods

### Patients

Twenty-five patients referring to the Eye Clinic of the Second University of Naples (Italy) met the clinical diagnostic criteria for BBS according to Beales et al. [7]. The female/male ratio was 2:3 and mean age was 25.6 years (range: 9–65 years). All procedures were conducted according to international guidelines and to the tenets of the Helsinki Declaration 2008 and 2013. Each patient (or parent or legal guardian) gave written consent to undergo DNA analysis, which was performed according to the guidelines for genetic testing approved by the Ministero della Salute, Rome, Italy (G.U. n. 224, 23th September 2004).

### Molecular study

Genomic DNA was extracted from peripheral blood leukocytes with the automated MagNA Pure LC system (Roche Diagnostics, Milan, Italy). DNA samples were first analyzed with the BBS–ALMS1 mutation array (Asper Biotech, Tartu, Estonia) that detects 253 sequence variants in the *BBS1-7*, *BBS9*, *BBS10*, *BBS12* and *ALMS1* genes. All exons and flanking intronic sequences of the *BBS1*, *BBS2* and *BBS10* genes were amplified with M13-tailed primer pairs and fully sequenced with M13 primers by using the Big Dye™ Terminator v.3.1 Sequencing kit and the ABI Prism 3730 DNA Analyzer (Applied Biosystems-Life Technologies Italia, Monza, Italy). Mutation numbering is based on the genomic and transcript reference sequences of *BBS1* (NG_009093.1, NM_024649.4), *BBS2* (NG_009312.1, NM_031885.3) and *BBS10* (NG_016357.1, NM_024685.3).

To predict the impact of the novel sequence variants on the expression of *BBS1*, *BBS2* and *BBS10*, we used the online tools Variant Effect Predictor (VEP) [14] and MutationTaster [15] that predict the effect of known and new variants (single nucleotide polymorphisms [SNPs], insertions, deletions, copy number variations or structural variants) on genes, transcripts, and protein sequences, as well as on regulatory regions. In the case of missense changes, VEP also assigns scale-invariant feature transform (SIFT) and polymorphism phenotyping v2 (PolyPhen-2) probability scores of the pathogenetic effect on the putative protein variant. VEP and MutationTaster not only handle single amino acid substitutions, but also insertions and deletions; they also identify non-canonical splice sites.

### Ophthalmological study

All 25 patients underwent a complete ophthalmological examination including best-corrected visual acuity (BCVA) measured using the Snellen chart, slit-lamp anterior segment examination, fundus examination, fundus photography, Goldmann visual field examination, standard ERG and optical coherence tomography (OCT). The ERG was recorded with a Ganzfield stimulator following the guidelines of the International Society of Clinical Electrophysiology of Vision [16]. OCT was performed with new generation tomography, which uses spectral domain-based techniques that allow the acquisition scans or 5 linear or a retinal area of 6 × 6 mm^2^ through 512 (horizontal) × 128 (vertical) scans (SD-OCT, Cirrus HD OCT, Carl Zeiss, Dublin, CA, USA).

### Renal study

Renal function was evaluated in 21 patients. Glomerular function was evaluated by estimating the GFR and the urine albumin-to-creatinine ratio (ACR). Albumin was measured in the early morning urine sample with a standard immunochemical method and expressed as urine ACR (mg/g). GFR was estimated according to the Modification of Diet In Renal Disease study group and the Chronic Kidney Disease Epidemiology Collaboration (CKD-EPI) [17], using standardized plasma creatinine measurement. In children, GFR was estimated with the Schwartz formula: GFR [(mL/min/1.73 m ^2^) = k × height (cm)/serum creatinine (mg/dL)]. The normal estimated GFR (eGFR) is 80–120 mL/min/1.73 m^2^; eGFR < 90 mL/min/1.73 m^2^ indicates impaired renal function and eGFR < 60 mL/min/1.73 m^2^ represents an increased risk factor for an adverse renal outcome. Moreover, to track longitudinal changes in GFR, we assessed eGFR 3 years after the first visit in patients with biallelic mutation of *BBS1* and *BBS10*. Tubular function was analyzed in patients with eGFR > 60 ml/min/1.73 m^2^. Acid-base balance was evaluated on arterial blood; urinary and plasma electrolytes were also measured. Renal concentrating ability was assessed by measuring urine osmolality 12 h after water restriction (Osmometer model 3320, A. De Mari, Italy). Urine osmolality < 750 mOsm/kg indicates a defect in urine concentrating capability. Renal morphology was determined by ultrasound.

### Audio-vestibular study

Six genotyped patients (5 men and 1 woman) aged between 13 and 38 years underwent audio-vestibular function testing. Information about drug history, pre- peri- and post-partum problems, previous audiological disorders, head trauma or neurological defects (e.g., history of migraine, epilepsy, vertigo) was obtained for all patients.

The hearing threshold was evaluated by liminal pure tone audiometry and assessment of perception by verbal speech audiometry. Subjects with a hearing threshold > 20 dB hearing level on the middle and/or high frequencies were considered to have hearing loss. The impedance analysis to evaluate the middle ear functioning was performed according to the guidelines of the American Speech-Language-Hearing Association. The presence of inner ear damage was verified by means of distortion product otoacustic emission (DPOAE). DPOAE was measured with a Madsen Cappella instrument, which generates two primary frequency tones, 2f1 and 2f2, with a stimulus frequency separation of f1/f2. Intensity of the custom stimulus was 40 dB sound pressure level (SPL) at both frequencies. The DPOAE was recorded by automatic scanning of the 250–8000 Hz frequency interval focused on the pure tone audiometric test frequencies. Auditory evoked potentials were evaluated with standard parameters. Three chloride silver electrodes were located in the vertex (active), mastoid (right or left) and forehead (ground) positions. Electrode impedances were maintained at ≤ 7 kΩ. Stimuli for auditory brainstem response recording were digitized at a rate of 20 kHz, and presented over headphones. Stimuli were 100 μs clicks presented monaurally at 110 dB SPL. Broadband noise at 70 dB SPL was presented to the opposite ear to mask any stimulation via acoustic cross talk. Clicks were presented at a rate of 21/s in 4-min runs. A conventional method of alternating click stimulus polarity was used to reduce stimulus artifacts in the average waveforms. Band-pass filtered the signals between 30 and 3000 Hz. The average waveform was focused on a period extending from 10 ms before the stimulus to 10 ms after the stimulus. Vestibular function was evaluated with statokinetic tests (Romberg, sensitized Romberg), detection of spontaneous nystagmus with video nystagmography, detection of evoked nystagmus with the head shaking test (HST) and the bithermal caloric test. The latter was performed with the Fitzgerald Hallpike method [18]. The ears were stimulated by irrigation with hot water (44 °C) and cold (30 °C) water. Patients were placed in a supine position with head flexed forward 30°, so that the straight line that joins the tragus to the outer canthus of eye was vertical. Irrigation was carried out with 250 ml of water at a flow rate about 5–8 ml/s for 40 s. The nystagmic reaction was induced in a few seconds and reached the maximum about 60 s after stimulation.

## Results

We enrolled 25 patients from 24 unrelated families. In addition to retinitis pigmentosa (RP), the main features of our patients that were consistent with the clinical diagnosis of BBS were postaxial polydactyly (21/25 patients), obesity (15/25 patients), a history of obesity (2/25 patients), renal abnormalities (18/21 patients). We found that 8/15 males had a history of hypogonadism, four of whom had cryptorchidism; 4/10 females had congenital abnormalities of the urogenital tract. Moreover, 18/25 patients showed intellectual disabilities including delay in learning development during early childhood and the need for educational support teachers. Consanguinity or presumed consanguinity (geographic isolates) was reported in 24% of patients (6/25).

All 25 patients underwent molecular analysis. First, we applied the BBS–ALMS1 mutation array, which revealed known sequence variants in 6/25 patients. These mutations affected the *BBS1*, *BBS2* and *BBS10* genes; therefore, we sequenced these genes in patients with one or no array-detected mutation. Sequencing confirmed the array data and revealed 12 additional sequence changes. Overall, our analysis revealed at least one sequence variant in 15/25 patients (60%). We found that 12/25 patients (about 48%) had biallelic putative disease-causing variants, which supported the clinical diagnosis.

**Fig. 1:**
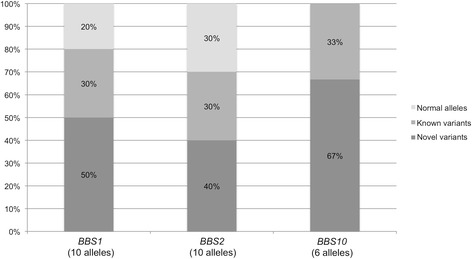
Prevalence of known vs the novel *BBS1*, *BBS2*, *BBS10* alleles in our genotyped BBS patients. Bars indicate, for the three groups of patients (*BBS1*, *BBS2*, *BBS10*), the percentage of independent alleles with new (*dark grey*), known (*intermediate grey*), normal alleles (*light grey*)

Table 1 illustrates the molecular data. Seven unrelated patients have sequence variants in *BBS1*; 5 of them have biallelic variants. Of the 8 independent *BBS1* variant alleles, 4 have sequence changes not previously linked to BBS. In summary, among the 10 independent *BBS1* alleles that we sequenced in the patients with *BBS1* variants, 30% carry known sequence variants, 50% carry sequence changes not previously linked to BBS, and 20% are normal (Fig. 1). The patient who carries the monoallelic p.V568M missense change in *BBS1* also has new biallelic pathogenic variants in *BBS2*. Overall, 5 patients carry *BBS2* sequence variants, but only 2 have a *BBS2* genotype (biallelic variants) that is consistent with the syndrome. In fact, two other patients have *BBS2* monoalleic variants, i.e., the new c.2144G > A (p.R715Q) and the c.986 T > C (p.M329T) (rs201146063) SNP, respectively, and no mutation in *BBS1* or *BBS10*. Another patient has a new monoallelic intronic sequence variant in *BBS2* (c.535-79_90del) and also biallelic, likely pathogenic variants in *BBS10*. In summary, among the 10 independent *BBS2* alleles that we sequenced, 30% carry known sequence variants, 40% carry sequence changes not previously linked to BBS, and 30% are normal (Fig. 1). Overall, 4 patients have biallelic pathogenic variants in *BBS10* (about 16%). We found that 4/6 (67%, Fig. 1) independent *BBS10* alleles carry new sequence variants, i.e., c.641 T > A (p.V214E), c.235dupA (p.T79Nfs*17), c.962A > G (p.Y321C) and c.2137_2140del (p.K713Ffs11*724Iext*1). Except for one case (Table 1, P.9), our homozygous BBS patients have consanguineous parents. In all cases, Mendelian segregation of the variant alleles was confirmed in parents and, when available, in unaffected siblings.

**Table 1:** Sequence variants identified in the BBS patients who tested positive to the molecular analysis

Patient ID		Genotype	
	*BBS1*	*BBS2*	*BBS10*
P.1^a^	**c.664G>C/c.664G>C (p.G222R)**	N/N	N/N
P.2^a^	**c.664G>C/c.664G>C (p.G222R)**	na	na
P.3^a,b^	c.1169T**>**G/c.1169T**>**G (p.M390R)	na	na
P.4^a,b^	c.1169T**>**G / c.1169T**>**G (p.M390R)	na	na
P.5^a^	c.1169T**>**G / c.1169T**>**G (p.M390R)	na	na
P.6	c.1169T**>**G/**c.1642delC** (p.M390R/p.L548Wfs*31)	na	na
P.7	**c.592-59G>A**/N	N/N	N/N
P.8	**c.1702G>A/N (p.V568M)**	**c.84delC/c.1059dupT** (p.P29Rfs*50/p.N354X)	N/N
P.9	N/N	c.225T**>**G/c.225T**>**G (p.V75G)	N/N
P.10	N/N	**c.2144G>A/N (p.R715Q)**	N/N
P.11	N/N	c.986T**>**C/N (p. M329T)	N/N
P.12	N/N	**c.535-79_90del**/N	c.2137_2140del/c.962A>G (p.K713Ffs*16/p.Y321C)
P.13	na	na	**c.235dupA**/c.271dupT (T79Nfs*17/p.C91LfsX5)
P.14^a^	na	na	c.509T**>**C/c.509T**>**C (p.L170S)
P.15^a^	N/N	N/N	**c.641**T**>A/c.641**T**>A (p.V214E)**

In bold, variants not previously linked to the BBS phenotype. For cDNA numbering, +1 corresponds to the A of the ATG translation initiation codon, which is codon 1. Reference gene sequences were *BBS1* (NG_009093.1, NM_024649.4), *BBS2* (NG_009312.1, NM_031885.3), *BBS10* (NG_016357.1, NM_024685.3)

*N* gene-specific normal allele, *n.a.* not analyzed

^a^With consanguineous parents

^b^Patients 3 and 4 are siblings

Bioinformatic prediction indicated that all the new sequence variants in the coding regions of *BBS1*, *BBS2* and *BBS10* were very likely “disease-causing”, whereas the two intronic variants were possible polymorphisms (Additional file 1: Table S1). Notably, many of the variants we identified are listed in the SNP database (National Center for Biotechnology Information, NCBI), all with a minor allele frequency < 0.0001 (Additional file 1: Table S1); however, this is the first report that links them to the BBS phenotype.

### Genotype to ocular phenotype correlation

**Fig. 2:**
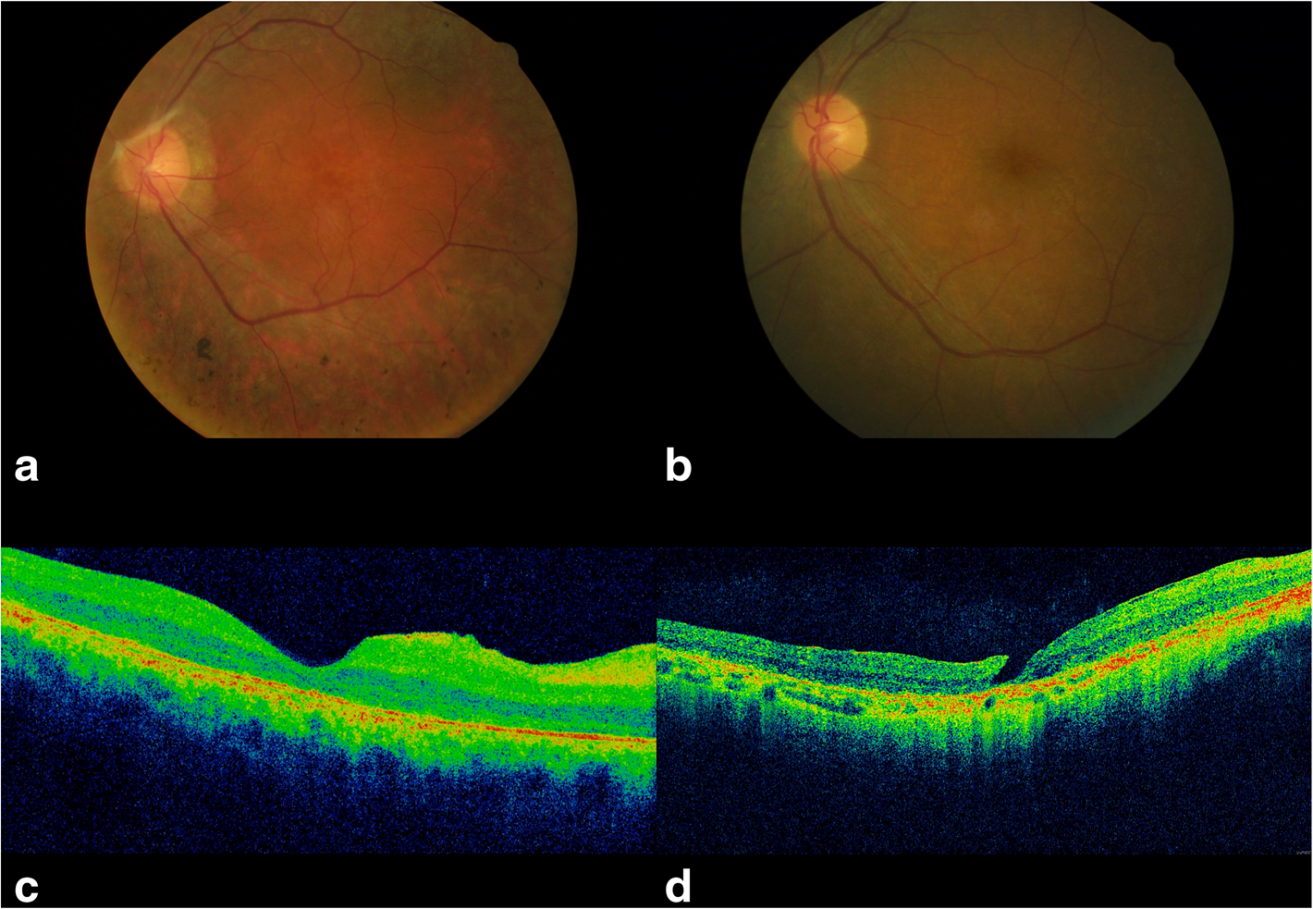
Representative images of the ocular findings in the BBS patients analyzed. Fundus photography showing **a** narrowing of retinal blood vessels, diffuse retinal pigment epithelial dystrophy with pigment clusters in mid-periphery; **b** narrowing of retinal blood vessels, widespread tapetoretinal degeneration and absence of pigment clusters. OCT scan showing **c** vitreomacular traction syndrome with retinal pigment epithelium dystrophy; (**d**) a macular lamellar hole with retinal pigment epithelium dystrophy

All patients with biallelic mutations (12/12) had visual defects (Table 2). Nine of these patients were affected by legal blindness having a visual acuity less than or equal to 20/200 (0.1 decimals); 3/12 patients had a visual acuity between 20/100 (0.2 decimals) and 20/70 (about 0.3 decimals). All 12 patients were able to perceive light. Visual impairment was not congenital, however 66.6% of patients had horizontal nystagmus, 58.3% strabismus and 50.0% had cataracts. At fundus examination, nine patients had osteoblast-like pigment clusters, mainly located on the equator, narrowing of retinal blood vessels, and optic disc pallor, which indicate diffuse retinal pigment epithelial dystrophy (Fig. 2a); three of them had macular dystrophy. In the remaining three patients, we observed widespread tapetoretinal degeneration and the absence of retinal pigment epithelium (Fig. 2b). All the 12 genotyped patients underwent ERG; they showed an extinguished, not age-related scotopic and photopic electroretinogram. Despite the nystagmus and fixation instability and low visual acuity, OCT images were acquired in nine patients, and these showed reduced macular thickness and retinal pigment epithelium dystrophy. Four patients showed an epiretinal membrane and three patients had signs of vitreomacular traction syndrome (Fig. 2c). One patient had a macular lamellar hole (Fig. 2d).

**Table 2:** Comparative genotype-phenotype correlation analysis in BBS patients

Patient ID	Phenotype											Genotype	
	Ocular			Renal			Audio-vestibular					Gene	Mutation
	Eye vision R/L	Fundus finding	Macular alterations	eGFR <60 ml/min/1.73 m^2^	Max uOsm <750 mOsm/kg	Ultrasound abnormalities	Hearing impairment	Timpanogram type	DPOAE L/R	Vestibular test	ABR alteration		
P.1	0.25/0.2	RP sp	Yes	No	Yes	Yes	No	AA	+/−	spontaneous NY	No	*BBS1*	c.664G>C/c.664G>C
P.2	0.3/0.2	RP sp	No	No	No	Yes	mixed	BB	+ /-	positional NY	only V wave	*BBS1*	c.664G>C/c.664G>C
P.3	0.2/0.1	RP	NP	NP	NP	NP	NP	NP	NP	NP	NP	*BBS1*	c.1169T>G /c.1169T>G
P.4	LP/LP	RP	NP	NP	NP	Yes	NP	NP	NP	NP	NP	*BBS1*	c.1169T>G /c.1169T>G
P.5	LP/LP	RP	Yes	No	No	Yes	No	AA	+/−	No	No	*BBS1*	c.1169T>G /c.1169T>G
P.6	0.05/0.03	RP	Yes	No	No	No	NP	NP	NP	NP	NP	*BBS1*	c.1169T>G/c.1642delC
P.8^a^	0.02/0.008	RP sp	Yes	No	Yes	Yes	NP	NP	NP	NP	NP	*BBS2*	c.84delC/c.1059dupT
												*BBS1*	c.1702G>A/N
P.9	0.02/0.02	RP	No	No	No	Yes	sensorineural	AA	−/−	positional NY	> latency I,III,V	*BBS2*	c.225T>G/c.225T>G
P.12	LP/LP	RP	Yes	No	Yes	Yes	NP	NP	NP	NP	NP	*BBS10*	c.2137_2140del/c.962A>G
												*BBS2*	c.535-79_90del/N
P.13	0.03/0.08	RP	Yes	Yes	Yes	Yes	sensorineural	AA	+/+	No	No	*BBS10*	c.235dupA/c.271dupT
P.14	LP/LP	RP	NP	NP	NP	NP	NP	NP	NP	NP	NP	*BBS10*	c.509T>C/c.509T>C
P.15	LP/LP	RP	Yes	No	Yes	Yes	sensorineural	AA	+/+	positional NY	No	*BBS10*	c.641T>A/c.641T>A

*F* female, *M* male, *R* right, *L* left, *RP* retinitis pigmentosa, *sp* sine pigmento, + partially present, − absent, *NY* nystagmus, *LP* light perception, *NP* not performed, *N* normal sequence

^a^Died of heart failure

Genotype-phenotype correlation revealed that the six patients with biallelic mutations in *BBS1* (aged 9 to 70 years) had a BCVA ranging from light perception to 0.3 decimals; four of them had a BCVA equal to or better than 1/10, three patients had nystagmus and five exotropia. Typical RP associated with subcapsular cataract was present in four patients, all over 25 years of age. The six *BBS1*-mutated patients presented extinguished scotopic and photopic ERG responses. OCT examination was performed only in four patients, three of whom showed vitreoretinal abnormalities (Table 2).

The two patients with biallelic mutations in *BBS2* had a BCVA ≤ 0.02 decimal and nystagmus; one of them also developed exotropia in the second decade of life. Both patients had diffuse retinal pigment epithelium dystrophy without cataract, and the oldest developed vitreoretinal abnormalities at OCT at the age of 19 years. In both cases, the scotopic and photopic ERG responses were extinguished.

We found that three of the four patients with biallelic variants in *BBS10* showed a BCVA of light perception; the remaining one had nystagmus associated with exotropia. All four showed typical RP; in the two eldest subjects (31 and 40 years old, respectively), subcapsular cataract also appeared at 30 years of age. Scotopic and photopic ERG responses were extinguished in all cases; OCT examination revealed vitreoretinal abnormalities in the three subjects we analyzed (Table 2). Despite the relatively small number of patients with a known genotype, genotype-phenotype correlation analysis revealed that, although BCVA reduction was age-related, *BBS1*-mutated patients had a significantly better visual acuity (*p* ≤ 0.006), with a slower progression of BCVA reduction (0.03 decimals/year; *p* < 0.01), compared with our *BBS10*- and *BBS2*-mutated patients.

### Genotype to renal phenotype correlation

**Fig. 3:**
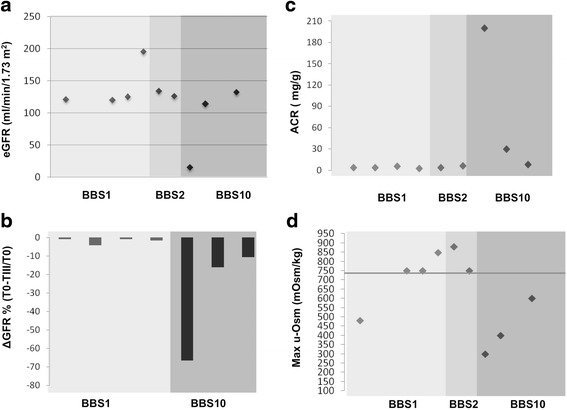
Analysis of renal function. **a** eGFR, calculated according to the CKD-EPI formula, in our BBS patients. The most severe renal dysfunction occurred in a patient carrying biallelic *BBS10* mutations. **b** Relative eGFR decline in 3 years. eGFR was estimated at baseline (T0) and after 3 years (TIII). Relative eGFR change (ΔGFR) during 3 years is expressed as percentage (%) of the T0. **c** Urine albumin-to-creatinine ratio (ACR). Two of three mutated patients showed ACR above 30 mg/g; the other patient showed normal ACR. **d** Maximal urine concentrating ability. Urine osmolality was measured in the second morning urine void, after overnight fasting and water restriction

We evaluated the renal phenotype in 9/12 BBS patients with a positive molecular test (Table 2). We obtained by telephone interview information about the renal functionality of the three patients who did not undergo renal examination. Interestingly, a 70-year-old woman, who was homozygote for the common *BBS1* mutation p.M390R (patient P.4, Table 2), underwent radical nephrectomy for a suspicious renal mass, 20 years earlier. Figure 3a correlates the patients’ eGFR with genotype. A young *BBS10* patient with congenital multicystic renal dysplasia, who was diagnosed with end-stage renal disease at the age of 23 years, had the lowest eGFR, and the eGFR reduction was associated to albuminuria and hypertension. The eGFR exceeded 90 ml/min/1.73 m^2^ in the other two *BBS10* patients, which however manifested tubular dysfunctions. In the patients with biallelic mutations in *BBS1* or *BBS2*, eGFR exceeded 90 ml/min/1.73 m^2^.

We also evaluated changes in eGFR in *BBS1* and *BBS10* patients (Fig. 3b) three years after baseline. The rate of decline (ΔGFR) correlated with GFR at baseline. In fact, renal dysfunction progressed faster in the patient with the lowest GFR. Interestingly, all patients with *BBS10* mutations had ΔGFR higher than 10%, whereas it was lower than 10% in the *BBS1*-mutated patients. The ACR, a marker of glomerular damage, exceeded 30 mg/g only in two patients, both with biallelic mutations in *BBS10* (Fig. 3c), whereas it was lower than 30 mg/g in our *BBS1* and *BBS2* patients.

Tubular function was evaluated only in subjects with an eGFR >60 ml/min/1.73 m^2^ (8/9 genotyped patients). The most common tubular dysfunction we found was a defect in urine concentrating ability, in agreement with a previous report [17]. All the three patients with biallelic mutations in *BBS10* had hyposthenuria even 12 h after water restriction (Fig. 3d) and therefore they were affected by renal dysfunction. In contrast, urine concentrating ability was normal in 3/4 patients with mutated *BBS1* and in both the *BBS2-*mutated patients (Fig. 3d).

Plasma electrolytes were within normal range in all patients (Additional file 1: Table S2). The mean concentrations of Na^+^ and K^+^ were normal, and Na^+^ and Cl^−^ fractional excretions (FE%) were lower than 1% in all patients except one with the *BBS10* mutation, who showed a slight increase in the fractional excretion of sodium, FENa^+^, and of chlorine, FECl^−^, (1.04 and 1.41%, respectively). Acid-base status was normal in all patients, except in one *BBS1* patient who was affected by metabolic acidosis. The urine Ca^+2^/creatinine (UCa/Cr) ratio was <0.2 mg/mg in all patients except one.

Abnormal kidney ultrasound appearance is a common feature of BBS patients [17]. Accordingly, in our cohort, 8/9 genotyped patients showed a broad range of kidney anatomical variations, i.e., parenchymal and peri-pelvic cysts, renal hypoplasia, pelvic dilation and fetal lobulations (Additional file 1: Table S3). We noted that the severity of structural abnormalities correlated with the severity of renal dysfunction.

### Genotype to audiovestibular phenotype correlation

Tonal audiometry analysis revealed cochlear sensorineural hearing loss (SNHL) in 3/6 patients (Additional file 1: Table S4); one had moderate bilateral SNHL, one mild bilateral SNHL, and one mild SNHL in the right ear. Another patient had a mixed moderate hearing loss more pronounced in the right ear. Hearing impairment in two patients required a hearing aid. Impedentiometry showed a type “A” normal tympanogram and normal stapedial reflexes in five patients (Table 2). However, at the Metz recruitment test, patient P.9 who had moderate SNHL had a considerably reduced dynamic range, i.e. the gap between the acoustic reflex threshold and the pure-tone audiometry hearing threshold level, which indicates cochlear pathology. The patient with mixed hearing loss showed a type “B” tympanogram and absent reflexes. All six patients had no otoacustic emission (Additional file 1: Table S5).

In the six patients we tested, speech audiometry headphones were normal; two patients had difficulty in verbal comprehension. No patient experienced true rotatory vertigo or dizziness during the vestibular test. Spontaneous nystagmus occurred in one patient. Positional nystagmus was observed in three patients (50%). In one patient, HST was positive and the caloric test showed a pathological caloric weakness in the right ear. The morphology and latency of the auditory brain response waves were normal in four patients. The latency of the I, III and V waves was increased in one patient, and only the V wave was observed at 80 dB hearing level in another patient.

## Discussion

Bardet-Biedl syndrome, a rare clinically and genetically heterogeneous disorder, is often misdiagnosed due to the high phenotypic variability and mainly because it shares several characteristic features with other ciliopathies [1]. Here we describe genetic and some clinical features of 12 BBS Italian patients and report the genotype-phenotype correlation.

Twenty-five Italian patients fulfilling the clinical criteria of BBS underwent molecular analysis of the *BBS1*, *BBS10* and *BBS2* genes because pathogenic variants in these genes have been found in 23%, 20% and 8% of BBS patients, respectively worldwide [13, 19]. We identified 21 independent alleles. Twelve families had biallelic genotypes that were consistent with the disease phenotype. Overall, we identified 17 different sequence variants, 6 known and 11 not previously linked to the disease, in about 60% of our patients, which indicates *BBS1*, *BBS2* and *BBS10* are frequently mutated in Italian patients. We also found monoallelic variants in *BBS1* and *BBS2* in two patients with biallelic pathogenic variants in *BBS2* and *BBS10*, respectively, which suggests the possibility of triallelism. We were unable to verify triallelic inheritance because these patients are the only affected members of their families. *BBS1* is the most frequently mutated gene in our cohort (28% of patients). Notably, two unrelated patients are homozygous for the p.G222R substitution in *BBS1*, which therefore represents 20% of *BBS1*-variant alleles in our cohort. The minor allele frequency reported in the ExAC database (Additional file 1: Table S1) indicates this variant is very rare worldwide. Therefore, p.G222R might be a founder disease allele, in Italy. Also p.M390R was frequent in our cohort of patients (30% of our *BBS1* variant alleles). Notably, the Exome Variant Server database records p.M390R in 0.25% (G = 23/T = 8567) of *BBS1* alleles of individuals of European-African descent. It has also been reported in approximately 80% of Caucasian *BBS1*-positive patients [19, 20]. Therefore, we cannot consider p.M390R a founder allele in Italy.

Surprisingly, we identified seven independent variant alleles in *BBS2* and only six in *BBS10*. However, whereas all the *BBS10*-mutated patients have genotypes fully consistent with the disease, only 2/5 *BBS2*-mutated patients have unambiguous pathogenic genotypes; the remaining three patients are heterozygous carriers of variants with a predicted low pathogenic significance (Additional file 1: Table S1).

Twelve of the variants we identified were not in the Human Gene Mutation Database (Version 2015.4) or in the Exome Variant Server. Five are point/subtle deletions or duplications that lead to a frameshift and/or premature stop codon, and therefore can be reasonably considered pathogenic. A further five are nucleotide substitutions that cause missense changes, and, in all cases, bioinformatic tools predicted that they exert potential pathogenic effects. Although these five substitutions are listed in the SNP database, they have not previously been found in BBS patients. The remaining two intronic sequence variants are predicted to be possible polymorphisms. In this context, segregation of the new variant alleles we found in the “biallelic” families strongly supports their pathogenic role.

In the two patients with potential triallelism [21], the clinical picture of the patient with biallelic mutations in *BBS2* and one putative pathogenic missense change in *BBS1* was particularly serious; in fact, this patient was born with a severe congenital aortic stenosis. This finding, which is unusual in BBS, negatively affected prognosis and caused the patient’s death at the age of 18 years. Since we limited our genetic analysis to three genes, we cannot exclude that other genotyped patients may have mutations in other genes that could exert an epistatic effect. In particular, two adult siblings (P.3 and P.4 in Tables 1 and 2) homozygous for the common *BBS1* variant p.M390R have different BBS phenotypes. This finding could be consistent with triallelism, also based on reports that some homozygotes for the p.M390R variant may or may not manifest the disease [20, 21]. However, it is noteworthy that the elder of our two siblings was the most severely affected and that BBS has an age-dependent penetrance and variable expressivity [22].

Ophthalmologic analysis revealed that visual acuity was age-related in the 12 genotyped patients. In fact, the visual defect was more severe in patients over 19 years of age (visual acuity ≤ 20/200) than in children (visual acuity between 20/100 and 20/70). No patient lost the ability to perceive light. Genotype-phenotype correlation indicated a severe reduction in BCVA in all patients except the two *BBS1*-mutated children probably because of their young age. Also the appearance of fundus abnormalities correlated with age. In fact, pigment-type osteoblasts, narrowing of the retinal vessels and pallor of the optic disc occurred late in the disease, namely, at a mean age 35.8 years in 8/12 (75%) patients. Instead, a “salt and pepper” fundus, which is considered a harbinger of retinal disease, appeared at a mean age of 15 years in 4/12 (25%) patients. In most cases, macular changes started in the early teens, whereas bone spicule pigments occurred mainly in early adulthood.

The scotopic and photopic components of ERG were altered in all the 12 genotyped patients. Eight of these patients (75%) underwent OCT study, which confirmed that the most frequent findings were outer retina thinning in the macular region and dystrophy of the pigmented epithelium [5]. Unlike a previous report [23], loss of “lamination” of the retina was not gene-related in our cohort. The retinal structure abnormalities in our patients did not correlate with genotype, age or disease severity.

We previously reported that the prevalence of vitreoretinal abnormalities in BBS is twice that in RP patients [24]. The most frequent abnormalities in RP patients were cystoid macular edema (20%) followed by epiretinal membrane (16%); vitreo-macular traction was reported in only 5% of patients [24]. Forty-four per cent of our BBS patients show an epiretinal membrane and 33.3% vitreo-macular traction, while no patient had cystoid macular edema.

Renal dysfunction was frequent in our cohort. The four *BBS1*-mutated patients we analyzed had mild renal abnormalities, normal eGFR and a normal electrolyte balance. One patient was affected by chronic metabolic acidosis. The ultrasound appearance of the kidney was unremarkable in one patient, while the others showed fetal lobulation or isolated parenchymal cysts. Kidney size and cortical thickness were normal in all patients of this *BBS1* subgroup. Only one *BBS1* patient had hyposthenuria. Modifier genes may have contributed to the onset of this dysfunction, which however is the most frequent renal dysfunction in BBS.

The two *BBS2-*mutated patients had a normal eGFR and tubular function, and a mild renal phenotype, similar to the *BBS1*-mutated patients. However, the ultrasound renal appearance differed greatly between the two groups of patients. The former had typical pelvic dilation/peripelvic cysts, whereas the latter had only mild fetal lobulation.

The three *BBS10*-mutated patients we analyzed have renal dysfunction. The most severely affected patient has two frameshift mutations, both leading to a non-functional protein. He was born with a renal malformation and was diagnosed with end stage renal disease at the age of 23 years. The other two *BBS10*-mutated adults have normal eGFR. However, one has a high ACR, which is a marker of glomerular damage. Both these *BBS10*-mutated patients had defective urine concentrating ability and a normal eGFR. We recently reported that *BBS10* knock-down affected forskolin-dependent AQP2 trafficking to the apical membrane of epithelial tubular cells, thus providing a potential explanation for hyposthenuria [25]. In addition, our finding that the decline of eGFR in three years was more severe in *BBS10*-mutated patients than in *BBS1*-mutated patients indicates that *BBS10* deficiency is related to a poor renal prognosis [26]. Therefore, we conclude that, in our cohort of patients, *BBS10* variant alleles are associated to severe kidney dysfunction.

The audiological study revealed that two *BBS10*-mutated patients and one *BBS2*-mutated patient had cochlear SNHL. In contrast, our *BBS1*-mutated patients have no or a mixed hearing impairment. These results differ from previous reports that hearing loss, mainly due to conductive loss, is a minor sign of BBS [8]. Two of our patients, affected by sensorineural and mixed hearing loss, respectively, were successfully treated with hearing aids. Therefore, patients with suspected BBS should undergo hearing evaluation. In addition, our DPOAE results shed light on the BBS phenotype. In fact, because all our BBS patients had abnormal DPOAEs, we concluded that they had alteration of outer hair cell function. This agrees with evidence that the BBS phenotype arises from a ciliary dysfunction and consequently it would affect tissues in which hair cells are present. Therefore, DPOAEs may be useful for the early detection of cochlear damage in BBS patients.

Vestibular function analysis suggested abnormalities in the nystagmographic framework in 4/6 patients (67%), which could be explained by the sharp decline in visual acuity. In one case, we hypothesized the presence of a unilateral peripheral vestibular lesion. Notably, two young patients have the same *BBS1* genotype (homozygous p.G222R), but very different hearing phenotypes even though they are of about the same age. Also in this case, the variable expressivity of BBS could reflect an epistatic effect of a putative triallelism.

## Conclusion

In our cohort of Italian BBS patients there is a high prevalence of RP with early onset of visual impairment, a high prevalence of renal dysmorphism and dysfunction, and of subclinical hearing defects that, although generally poorly substantiated, are a useful hallmark of BBS. *BBS1*, *BBS2* and *BBS10* are major causative genes also in Italian BBS patients and the identification of new mutations demonstrates a high allelic heterogeneity. Pathogenic variants of *BBS10* correlated with a worse outcome, at least in terms of renal, ocular and audiovestibular phenotypes. As *BBS10* variants severely affect renal structure and function, patients manifesting kidney malformation should be scanned for mutations of this gene. Overall, our study may help to improve the identification of this complex disorder.

## Additional file

Additional file 1: Table S1. Bioinformatically-predicted putative effects of the variants linked to the BBS phenotype. **Table S2** Electrolytes and acid base balance in BBS patients. **Table S3** Correlation between renal structural alterations and genotype in BBS patients. **Table S4** Results of pure tone audiometry. **Table S5** DPOAE results. (DOCX 94 kb)

## Abbreviations

ACR: Albumin-to-creatinine ratio
BBS: Bardet-Biedl syndrome
BCVA: Best-corrected visual acuity
CKD-EPI: Chronic Kidney Disease Epidemiology Collaboration
DPOAE: Distortion product otoacustic emission
eGFR: Estimated GFR
ERG: Electroretinogram
ExAC: Exome Aggregation Consortium
FECl: Fractional excretion of chlorine
FENa: Fractional excretion of sodium
GFR: Glomerular filtration rate
OCT: Optical coherence tomography
PolyPhen: Polymorphism phenotyping
RP: Retinitis pigmentosa
SIFT: Scale-invariant feature transform
SNHL: Sensorineural hearing loss
SNP: Single nucleotide polymorphism
UCa/Cr: Urine Ca^+2^/creatinine
VEP: Variant Effect Predictor
