# Supplementary material for: Genetic characterization of Italian patients with Bardet-Biedl syndrome and correlation to ocular, renal and audio-vestibular phenotype: identification of eleven novel pathogenic sequence variants
Source: BMC Med Genet. 2017 Feb 1;18:10. doi: 10.1186/s12881-017-0372-0 (PMC5286791; doi:10.1186/s12881-017-0372-0)
Supplement: Additional file 1: Table S1. — Bioinformatically-predicted putative effects of the variants linked to the BBS phenotype. Table S2 Electrolytes and acid base balance in BBS patients. Table S3 Correlation between renal structural alterations and genotype in BBS patients. Table S4 Results of pure tone audiometry. Table S5 DPOAE results. (DOCX 94 kb) [file 12881_2017_372_MOESM1_ESM.docx]

**Supplementary Tables**

**Table S1. Bioinformatically-predicted putative effects of the variants linked to the BBS phenotype**

**Gene Mutation SNP RefSeq MAF_ExAC_0.3 MAF_EVS VEP SIFT/PolyPhen MutationTaster Possible effect**

*BBS1* c.592-59G>A -- -- likely not pathogenic --

c.664G>C rs761760689 0.00001650 -- Moderate deleterious/possibly damaging likely pathogenic protein features affected (p.G222R)/splice site changes

c.1642delC High -- pathogenic frameshift/NMD/truncated protein (p.L548Wfs*31)

c.1702G>A rs754300140 0.00000826 -- Moderate deleterious/ possibly damaging likely pathogenic protein features affected (p.V568M)

*BBS2* c.84delC High -- pathogenic frameshift/NMD/truncated protein (p.P29Rfs*50)

c.535-79_90del -- -- likely not pathogenic --

c.986T>C rs201146063 0.00006589 -- Moderate tolerated/benign likely pathogenic protein features affected (p.M329T)/splice site changes

c.1059dupT High -- pathogenic NMD/splice site changes/frameshift (p.N354X)

c.2144G>A rs761068592 0.00003295 -- Moderate tolerated/benign likely pathogenic amino acid changed (p.R715Q)/splice site changes

*BBS10* c.235dupA rs760693838 0.00001647 -- High -- pathogenic frameshift/NMD/splice site changes/frameshift (T79Nfs*17)

c.641T>A Moderate deleterious/ possibly damaging likely pathogenic protein features affected (p.V214E)/splice site changes

c.962A>G rs771325212 0.00000824 -- Moderate deleterious/ possibly damaging likely pathogenic protein features affected (p.Y321C)/splice site changes

c.2137_2140del High -- pathogenic prolonged protein (p.K713Ffs11*724Iext*1)/splice site changes

Reference gene sequences: *BBS1* (NG_009093.1, NM_024649.4), *BBS2* (NG_009312.1, NM_031885.3), *BBS10* (NG_016357.1, NM_024685.3). For cDNA numbering, +1 corresponds to the A of the ATG translation initiation codon, which is codon 1. MAF, minor allele frequency. VEP, variant effect predictor. Deleterious impact grading of variants by VEP is also based on SIFT and PolyPhen prediction (for missense changes).

**Table S2. Electrolytes and acid base balance in BBS patients**

| **Mutated gene** |  | ***BBS 1*** | | | |  | ***BBS2*** | |  | ***BBS10*** | |
| --- | --- | --- | --- | --- | --- | --- | --- | --- | --- | --- | --- |
| **Patient ID** |  | **P.1** | **P.2** | **P.5** | **P.6** |  | **P.8** | **P.9** |  | **P.12** | **P.15** |
| Serum Na ^+^ (mEq/L) | n.v. 135-145 | 141 | 142 | 144 | 145 |  | 141 | 142 |  | 141 | 145 |
| Serum K ^+^ (mEq/L) | n.v. 3.5 - 5.2 | 4.5 | 4.2 | 3.9 | 4.3 |  | 4.4 | 4.5 |  | 4.1 | 4.1 |
| Arterial plasma pH | n.v. 7.35–7.45 | 7.40 | 7.41 | 7.40 | **7.31** |  | 7.44 | 7.45 |  | 7.43 | 7.40 |
| Arterial plasma HCO_3_^-^ (mmol/L) | n.v. 21–27 | 25 | 24 | 25.3 | **20.2** |  | 24 | 25 |  | 24 | 24 |
| FeNa^+^(%) | n.v. < 1% | 0.80 | 0.66 | 0.54 | 0.64 |  | 0.70 | 0.67 |  | 0.51 | **1.04** |
| FeCl^-^ (%) | n.v. < 1% | 0.94 | 0.80 | 0.72 | 0.78 |  | 0.82 | 0.90 |  | 0.68 | **1.41** |
| uCa/Cr (mg/mg) | n.v. <0.21 | 0.07 | 0.12 | 0.03 | 0.11 |  | 0.08 | 0.11 |  | 0.09 | **0.51** |

n.v., normal values. In bold, abnormal findings.

**Table S3. Correlation between renal structural alterations and genotype in BBS patients**

| **Mutated gene** | ***BBS1*** | | | |  | ***BBS2*** | |  | ***BBS10*** | | |
| --- | --- | --- | --- | --- | --- | --- | --- | --- | --- | --- | --- |
| **Patient ID** | **P.1** | **P.2** | **P.5** | **P.6** |  | **P.8** | **P.9** |  | **P.12** | **P.13** | **P.15** |
| Fetal lobulations | + | - | - | - |  | - | + |  | + | + | - |
| Parenchymal cysts | - | - | + | - |  | - | - |  | - | + | + |
| Cysts/diverticula/dilatation of the calyces | + | + | - | - |  | + | - |  | - | + | + |
| Hypoplasia | - | - | - | - |  | - | - |  | - | + | - |
| No abnormalities | - | - | - | + |  | - | - |  | - | - | - |

**Table S4. Results of pure tone audiometry in the six BBS patients who underwent the test**

| **Patient ID** | Left ear | | | | | | | | |  | Right ear | | | | | | | | |
| --- | --- | --- | --- | --- | --- | --- | --- | --- | --- | --- | --- | --- | --- | --- | --- | --- | --- | --- | --- |
|  | 125 Hz | 250 Hz | 500 Hz | 1 kHz | 2 kHz | 3 kHz | 4 kHz | 6 kHz | 8 kHz |  | 125 Hz | 250 Hz | 500 Hz | 1 kHz | 2 kHz | 3 kHz | 4 kHz | 6 kHz | 8 kHz |
| P.1 | 15 | 15 | 15 | 15 | 15 | 15 | 15 | 15 | 15 |  | 15 | 15 | 15 | 15 | 15 | 15 | 15 | 15 | 15 |
| P.2 | 40 | 40 | 30 | 30 | 20 | 20 | 30 | 40 | 50 |  | 55 | 55 | 50 | 55 | 40 | 50 | 50 | 50 | 60 |
| P.5 | 15 | 15 | 15 | 5 | 5 | 5 | 10 | 15 | 15 |  | 0 | 0 | 0 | 5 | 5 | 0 | 5 | 10 | 5 |
| P.9 | 10 | 15 | 20 | 25 | 40 | 45 | 50 | 45 | 45 |  | 10 | 15 | 20 | 40 | 40 | 45 | 50 | 40 | 45 |
| P.13 | 20 | 10 | 10 | 25 | 10 | 15 | 5 | 15 | 10 |  | 15 | 15 | 15 | 40 | 45 | 45 | 60 | 40 | 35 |
| P.15 | 20 | 20 | 20 | 20 | 20 | 20 | 25 | 35 | 20 |  | 20 | 20 | 20 | 20 | 20 | 20 | 25 | 35 | 20 |

**Table S5. DPOAE results in the six BBS patients who underwent the test**

|  | **DPOAE** | | | | | | | | | | | | |
| --- | --- | --- | --- | --- | --- | --- | --- | --- | --- | --- | --- | --- | --- |
| **Patient ID** | 1 kHz  LE | 2 kHz LE | 3 kHz LE | 4 kHz LE | 6 kHz LE | 8 kHz LE |  | 1 kHz RE | 2 kHz RE | 3 kHz RE | 4 kHz RE | 6 kHz RE | 8 kHz RE |
| P.1 | p | p | p | p | a | a |  | p | p | p | p | p | p |
| P.2 | a | a | p | p | a | a |  | a | a | a | a | a | a |
| P.5 | p | p | p | a | a | a |  | p | p | p | p | a | a |
| P.9 | a | a | a | a | a | a |  | a | a | a | a | a | a |
| P.13 | p | p | a | p | a | a |  | p | p | a | a | a | a |
| P.15 | nd | p | p | p | a | a |  | nd | p | p | p | a | p |

LE, left ear; RE, right ear; p, present; a, absent; nd, not done.
